# Case Report: Case report: A rare case of middle-ear Rhabdomyosarcoma in a 4-year-old boy

**DOI:** 10.12688/f1000research.20558.2

**Published:** 2019-11-15

**Authors:** Richard Menzies-Wilson, Gentle Wong, Prodip Das

**Affiliations:** 1 Urology Department, Whipps Cross University Hospital, London, UK; 2 Ear, Nose and Throat Department, Brighton and Sussex University Hospitals, Brighton, UK

**Keywords:** Rhabdomyosarcoma, middle ear

## Abstract

We present a rare case of a four-year-old boy with a botyroid embryonal rhabdomyosarcoma of the right middle ear. Rhabdomyosarcoma is a soft tissue malignancy which is thought to originate from embryonic mesenchymal cells of striated skeletal muscle.  It is a disease primarily of children and is exceptionally rare in parameningeal regions.  The diagnosis is often delayed and easily misdiagnosed as aural polyp. Therefore, advanced disease is common at the time of diagnosis.  A four-year-old boy presented with a four-month history of recurrent left ear blood and pus discharge, otalgia and fevers. He attended his GP three times and paediatric A&E 13 times where he received antibiotics for presumed otitis media and externa. He was eventually referred to the otolaryngology department and underwent an examination under anaesthesia of ear and excisional biopsy of a suspicious aural polyp.  Staging chest CT and PET scan showed no loco-regional spread or distal metastasis. Magnetic resonance imaging demonstrated absence of invasion into adjacent organs.  Histology confirmed embryonal rhabdomyosarcoma, botryoid subtype.  Subsequent to the initial excision of the polyp, he was started on an ifosfamine, vincristine and actinomycin (IVA) chemotherapy regime in three weekly cycles for nine cycles with concomitant radiotherapy. Two weeks subsequent to his first chemotherapy dose he presented with a House-Brackmann II-III facial nerve palsy but no other middle ear complications. He was started on intravenous antibiotics and dexamethasone. The facial nerve palsy incompletely resolved with treatment.

## Introduction

Rhabdomyosarcoma is a rare soft tissue malignancy with unknown aetiology. It is thought to originate from embryonic mesenchymal cells of striated skeletal muscle. It is a disease primarily of children, with two-thirds of patients presenting under the age of six years ^1^. The commonest areas of the body to be affected are the head and neck, the bladder and reproductive organs. They are rare elsewhere and are exceptionally rare in parameningeal regions, including the ear and mastoid.

Symptoms depend on the location of the tumour. Aural involvement often has non-specific symptoms such as fever and otalgia. If the tumour is significant in size and protrudes through the tympanic membrane, then otorrhoea and hearing loss may result. Rarely, facial nerve palsy may occur. The diagnosis is often delayed and easily misdiagnosed as aural polyp. Therefore, advanced disease is common at the time of diagnosis.

We present a rare case of a four-year-old boy with a botyroid embryonal rhabdomyosarcoma of the right middle ear and review the literature.

## Case report

A four-year-old boy presented with a four-month history of recurrent left ear blood and pus discharge, otalgia and fevers. He attended his general practitioner (GP) three times and Paediatric Accident & Emergency 13 times where he received oral, topical and intravenous antibiotics for presumed otitis media and externa. He had no relevant family history. He was eventually referred to the otolaryngology department after 19 months and was listed for an examination under anaesthesia of and excisional biopsy of a suspicious aural polyp 2 weeks after presentation to the otolaryngology department team. Intra-operatively, a large lesion extending from the middle ear through the tympanic membrane and into the external auditory canal was noted which was incompletely resected as only tissue lateral to the tympanic membrane was resected. The lesion was found to be highly vascular.

Histopathological findings were in keeping with embryonal rhabdomyosarcoma, botryoid subtype. This rare diagnosis required a second opinion from the Histology Department at the Royal Marsden Hospital. Immunochemical staining revealed sheets of rounded tumour cells with scant cytoplasm and inconspicuous nucleoli, which were distinguished by the formation of polypoid and grapelike tumour masses. Malignant cells in an abundant myxoid stroma were observed. Staining was positive for desmin and muscle-specific actin.

Magnetic resonance imaging (MRI) of the head was performed within two weeks of resection of the tumour, which demonstrated absence of invasion into adjacent organs. Staging chest MRI showed no evidence of metastases and PET scan showed no loco-regional spread or distal metastasis. Bone marrow trephine and CSF were negative. The patient’s haemoglobin levels were borderline anaemic but all routine blood tests were otherwise within normal limits.

At 5 weeks after initial presentation he was started on nine cycles of IVA chemotherapy: ifosfamine (3 g/m ^2^ on days 1 and 2); vincristine (1.5 mg/m ^2^ weekly during the first 7 weeks, then only on day 1 of each cycle); and Dactinomycin (at 1.5 mg/m ^2^ on day 1) in three weekly cycles with concomitant radiotherapy.

Two weeks subsequent to his first chemotherapy dose he was admitted with neutropenic sepsis and also presented with a House-Brackmann II-III facial nerve palsy but no other middle ear complications. He was started on paediatric doses of intravenous Piperacillin / tazobactam and gentamicin for 5 days as an inpatient prior to being discharged on a paediatric oral dose of ciprofloxacin for the remainder of his chemotherapy course. The facial nerve palsy incompletely resolved to a House-Brackmann I-II with a 2-week course of 3 mg dexamethasone twice daily. He is being followed up on the following schedule: 3–4 months in the first 2–3 years, then twice a year up to the fifth year. Disease free follow-up is 18 months.

## Literature review

We searched EMBASE, MEDLINE, UpToDate, CINAHL, TRIP Database, BMJ Best Practice, Google Scholar, Cochrane Database of Systematic Reviews and NICE Evidence Search with the following search terms: rhabdomyosarcoma (RMS); ear; aural; middle ear; inner ear; ear canal, parameningeal; ear neoplasm in July 2018.

Inclusion criteria were: diagnosis of rhabdomyosarcoma of the middle ear; published in a peer reviewed journal; any geographical location or language; any publication date; any age of patient.

In total 11 case reports were found. Of these, there was an equal male-female split. The clinical presentations were similar throughout the case reports, mimicking recurrent otitis media/externa. The average survival across the case reports was 7.2 months. We summarise the case reports in Table 1.

**Table 1.** Summary of case reports.

Case	Authors	Patient sex	Age	Journal (year)	Symptom	Investigation	Subtype	Management	Prognosis
1	Phatak *et al.*	M	2	Indian Journal of Otol 1999	CNVII palsy	CT	Embryonal	Surgery + CRT	Alive at 18 mo
2	Hu *et al.*	M	3	Otol HNS 2002	Otorrhoea	CT	Embryonal	Surgery + CRT	Alive at 18 mo
3	Hu *et al.*	M	33	Otol HNS 2002	CNVII palsy + Otalgia	CT	Embryonal	Surgery + CRT	Alive at 24 mo
4	Abbas *et al.*	M	3	ENT Jour 2005	Otorrhoea	CT	Embryonal	CRT	Died at 11 mo
5	Viswanatha	M	4	ENT Jour 2007	CNVII + CNVI	CT	Embryonal	Surgery + CRT	Died at 3 mo
6	Muranjan *et al.*	F	5	BMJ Case rep 2014	CNVII + Otorrhoea	CT + MRI	Anaplastic subtype of embryonal	CRT	Died at 5 mo
7	Vasiwala *et al.*	M	6	Med J Malaysia 2015	CNVII + Otorrhoea	CT	Embryonal	Surgery + CRT	Died at 7 mo
8	Bhargava *et al.*	M	31	J Surg Case Rep 2012	CNVII + Otorrhoea	CT	Embryonal	Surgery + CRT	Died at 6 mo
9	Salunke *et al.*	M	3	J Pediatr Neurosci 2012	CNVII + Otalgia + H/Loss	CT + MRI	Embryonal	Surgery + CRT	Died at 6 mo
10	Vegari *et al.*	F	3	Case Rep Otolary 2012	Otorrhoea	CT	Embryonal	Surgery	Died at 12 mo
11	King *et al.*	F	2	Brachytherapy 2017	Mass	CT + MRI	Botyroid	Chemo + Brachy	Alive at 8 mo

## Discussion

Rhabdomyosarcoma of the middle ear and mastoid is rare ^2^. Around 30% of cases of paediatric rhabdomyosarcoma occur in the head and neck involving the orbits, base of skull, nasal cavity and nasopharynx ^3^ but only 3% occur in the ear or mastoid ^4^. In total, 63% of rhabdomyosarcomas occur in children under 10 years old, with a peak between 2 and 5 years old ^5^.

Presentation often involves purulent or blood-stained discharge, hearing loss, aural polyps and granulation tissue. Clinical diagnosis is often delayed since the presentation is similar to that of chronic suppurative otitis media ^6^. By the time of diagnosis, a facial nerve palsy is usually present and there is involvement of both the middle and external ear and petrous bone ^7^. Metastases commonly spread to the lungs, liver, bones and extremities, and are present in approximately 30% of cases ^3^.

According to the International Classification of Rhabdomyosarcoma, these tumours are histopathologically classified into five categories: botryoid, spindle cell, embryonal, alveolar, and undifferentiated. Embryonal rhabdomyosarcoma is the most common, accounting for 70–80%, occurring mostly in the orbit and parameninges ^3^. Alveolar is the second most common, usually presenting in the deep soft tissues of the extremities ^4^.

Risk factors for rhabdomyosarcomas are poorly understood. Epidemiological studies have suggested a link with radiation and recreational drug *in utero*, lower socioeconomic status and use of antibiotics soon after birth ^8^. Several genetic syndromes, including some commonly known to otolaryngologists such as Rubinstein-Taybi syndrome and Beckwith-Wiedemann syndrome, are associated with increased prevalence of rhabdomyosarcoma.

Historically, rhabdomyosarcomas were invariably fatal. Now, 5-year survival rates of around 80% have been reported ^9^; however, surgical removal is often difficult in head and neck cases given the stage of the neoplasm and anatomy. Consequently, the mainstay of treatment is often chemoradiotherapy. Parameningeal tumours, especially of the middle ear and mastoid, have a poor prognosis because of the proximity to the brain ^6^. Of the categories, embryonal have the best prognostic factor and alveolar the worst and children have a better prognosis than adults ^4^.

## Conclusion

Rhabdomyosarcoma of the ear is a rare presentation but one which should be considered in young children with recurrent otitis media. If missed, facial nerve involvement is a common as well as local meningeal and distant metastasis. Multi-disciplinary involvement and patient education is mandatory in ensuring the highest survival rates. Treatment consists of a combination of surgery, chemotherapy and/or radiotherapy.

## Data availability

All data underlying the results are available as part of the article and no additional source data are required.

## Consent

Written informed consent for publication of their clinical details as obtained from the parent of the patient study.
