# Supplementary material for: Case Report: Case report: A rare case of middle-ear Rhabdomyosarcoma in a 4-year-old boy
Source: F1000Res. 2019 Nov 15;8:1734. Originally published 2019 Oct 9. [Version 2] doi: 10.12688/f1000research.20558.2 (PMC6823896; doi:10.12688/f1000research.20558.2)
Supplement: Peer review history

# Peer review: Reviewer response for version 1

**DOI:** 10.5256/f1000research.22599.r54909

**Published:** 2019-11-05

**Authors:** Adam Haymes

**Affiliations:** 1 Department of Otolaryngology Surgery, Medway Maritime Hospital, Gillingham, Kent, UK

This article describes a case of botyroid embryonal rhabdomyosarcoma of the right middle ear in a four year old child. It covers the clinical presentation, workup and subsequent management of this rare entity and reviews the pertinent literature of other case descriptions.

 The case report is very well written, informative, covers an interesting and unusual topic, and adequately covers all of the objectives set out in the guidelines. 

 The background of the case’s history and progression are described in excellent detail.

 Description of the physical examination and workup of the patient are detailed and methodical, as are the descriptions of diagnostic tests, treatments and outcomes.

 Emphasis is placed on the rarity of this clinical entity and thus it's contribution to the literature is a useful one. There were no novel treatments in this case report, however the description of the chemotherpay regimen will be useful for other clinicians. Description of the recurrent presentations to the GP and A&E departments are a sobering reminder of the need for these clinicians to recognise unusual clinical patterns, not rely on previous diagnoses and seek help from specialists early in such scenarios. 

 Images of the histology would have been a nice to see given the rarity of this clinical entity.

I confirm that I have read this submission and believe that I have an appropriate level of expertise to confirm that it is of an acceptable scientific standard.

# Peer review: Reviewer response for version 1

**DOI:** 10.5256/f1000research.22599.r54912

**Published:** 2019-10-31

**Authors:** Craig Hickson

**Affiliations:** 1 Leicester Royal Infirmary, Leicester, UK

This is a generally well written case report detailing a rare disease process in an unusual anatomical location. It covers pathogenesis, presentation, problems with diagnosis, treatment and prognosis.

 It has multiple grammatical errors which need review and correcting:

 'Recreational drugs in utero.'

 The diagnosis is often delayed and easily misdiagnosed as AN aural polyp

 'A four-year-old boy presented with a four-month history of recurrent left ear blood and pus discharge' - please revise to A four-year-old boy presented with a four-month history of recurrent haemo-purulent discharge from the left ear.

 In addition I have the following comments/suggestions:

 Intra- operatively, a large lesion extending from the middle ear through the tympanic membrane and into the external auditory canal was noted which was resected. The lesion was found to be highly vascular.

  Was it totally resected? Via a tympanotomy? Or was the tissue lateral to the TM resected. If a tympanotomy was performed what was the status of the ossicles?

 You say that the facial nerve palsy incompletely resolved. Please give details using House-Brackmann or other suitable classification.

 How do you arrive at '3% of paediatric rhabdomyosarcoma occurring in the head and neck involving the …………..'?

 There are only 11 cases reported. You would need 100/3 = 30 cases or more to be able to state this?

 Could intra operation images and high res CT image be included?

 Please state the disease free followup to date of your patient.

 Please comment on hearing outcome.

 There is no mention specific mention of MDT discussion? You state a second opinion was sought but surely this case went through an MDT?

I confirm that I have read this submission and believe that I have an appropriate level of expertise to confirm that it is of an acceptable scientific standard.

# Response

**Published:** 2019-11-11

**Authors:** Richard Menzies-Wilson

Many Thanks for your comments.  We have adjusted the manuscript to reflect your feedback.

# Peer review: Reviewer response for version 1

**DOI:** 10.5256/f1000research.22599.r54913

**Published:** 2019-10-16

**Authors:** Max Cunnane

**Affiliations:** 1 Royal Sussex County Hospital, Brighton, UK

This is an interesting, concise and well-constructed case report and review of the relevant literature surrounding rhabdomyosarcoma affecting the middle ear. This is a rare entity and as such additional case reports are valuable in adding to the existing literature.

 The case report contains adequate description of the patient and detail of the presentation and initial investigations, which would be in line with the reviewers own practice.

 The case highlights the need for high clinical suspicion when treating non-resolving recurrent ear infections in children which is a useful message for clinicians.

 The literature review results are presented clearly and contain all relevant information regarding the case’s reported. However, there is a lack of detail surrounding which chemotherapy/radiotherapy regimes were used. It is also unclear if they were adjuvant or primary treatment modalities for the other cases described.

I confirm that I have read this submission and believe that I have an appropriate level of expertise to confirm that it is of an acceptable scientific standard.
